# Tuberculosis contact tracing, Angola

**DOI:** 10.2471/BLT.23.290068

**Published:** 2024-01-31

**Authors:** Joan Martínez-Campreciós, Eva Gil, Sandra Aixut, Milagros Moreno, Adriano Zacarias, Arlete Nindia, Estevao Gabriel, Juan Espinosa-Pereiro, Adrián Sánchez-Montalvá, Maria Luisa Aznar, Israel Molina

**Affiliations:** a International Health Unit Vall d’Hebron-Drassanes, Infectious Diseases Department, Vall d’Hebron University Hospital, PROSICS Barcelona, Passeig de la Vall d’Hebron 119, 08035 Barcelona, Spain.; b Hospital Nossa Senhora da Paz, Cubal, Benguela, Angola.

## Abstract

**Objective:**

To assess the outcomes of a contact-tracing programme to increase the diagnosis of tuberculosis in Cubal, Angola and offer preventive treatment to high-risk groups.

**Methods:**

A health centre-based contact-tracing programme was launched in Hospital Nossa Senhora da Paz in March 2015 and we followed the programme until 2022. In that time, staffing and testing varied which we categorized as four periods: medical staff reinforcement, 2015–2017, with a doctor seconded from Vall d’Hebron University Hospital, Spain; routine staff, 2017–2021, with no external medical support; community directly observed treatment (DOT), 2018–2019 with community worker support; and enhanced contact tracing, 2021–2022, with funding that allowed free chest radiographs, molecular and gastric aspirate testing. We assessed differences in contacts seen each month, and testing and treatment offered across the four periods.

**Findings:**

Overall, the programme evaluated 1978 contacts from 969 index cases. Participation in the programme was low, although it increased significantly during the community DOT period. Only 16.6% (329/1978) of contacts had a chest radiograph. Microbiological confirmation increased to 72.2% (26/36) after including molecular testing, and 10.1% (200/1978) of contacts received treatment for tuberculosis. Of 457 contacts younger than 5 years, 36 (7.9%) received preventive tuberculosis treatment. Half of the contacts were lost to follow-up before a final decision was taken on treatment.

**Conclusion:**

Contact tracing increased the diagnosis of tuberculosis although engagement with the programme was low and loss to follow-up was high. Participation increased during community DOT. Community-based screening should be explored to improve participation and diagnosis.

## Résumé

**Objectif:**

Évaluer l'efficacité d'un programme de suivi des contacts pour améliorer le dépistage de la tuberculose à Cubal, en Angola, et proposer un traitement préventif aux groupes à haut risque.

**Méthodes:**

Un programme de suivi des contacts en centre de santé a été lancé en mars 2015 à l'Hospital Nossa Senhora da Paz, et nous l'avons accompagné jusqu'en 2022. Comme les effectifs et le dépistage ont évolué durant cette période, nous l'avons divisée en quatre phases: renforcement de l'équipe médicale entre 2015 et 2017, avec un médecin détaché de l'hôpital universitaire Vall d'Hebron, en Espagne; personnel régulier entre 2017 et 2021, sans assistance médicale externe; traitement sous surveillance directe (TSD) au niveau communautaire entre 2018 et 2019, avec l'aide des agents communautaires; et enfin, suivi renforcé des contacts entre 2021 et 2022, avec un financement permettant d'effectuer gratuitement des radiographies thoraciques, ainsi que des tests moléculaires et d'aspiration gastrique. Nous avons évalué les différences dans les contacts vus chaque mois, mais aussi dans les tests et traitements proposés durant les quatre phases.

**Résultats:**

Au total, le programme a examiné 1978 contacts issus de 969 cas indicateurs. La participation au programme était faible, bien qu'elle ait considérablement augmenté au cours de la phase de TSD au niveau communautaire. À peine 16,6% (329/1978) des contacts ont fait l'objet d'une radiographie thoracique. La confirmation microbiologique a atteint 72,2% (26/36) après avoir inclus les tests moléculaires, tandis que 10,1% (200/1978) des contacts ont reçu un traitement contre la tuberculose. Sur les 457 contacts âgés de moins de 5 ans, 36 (7,9%) se sont vus administrer un traitement préventif contre la tuberculose. La moitié des contacts ont été perdus de vue avant qu'une décision finale n'ait été prise en matière de traitement.

**Conclusion:**

Le suivi a permis de mieux diagnostiquer la tuberculose, malgré la faible participation au programme et le nombre élevé de contacts perdus de vue. La participation a augmenté pendant la phase de TSD au niveau communautaire. Le dépistage au niveau communautaire devrait être renforcé afin d'accroître le taux de participation et de diagnostic.

## Resumen

**Objetivo:**

Evaluar los resultados de un programa de rastreo de contactos para aumentar el diagnóstico de la tuberculosis en Cubal (Angola) y ofrecer tratamiento preventivo a los grupos de alto riesgo.

**Métodos:**

En marzo de 2015, se inició un programa de rastreo de contactos basado en centros sanitarios en el Hospital Nossa Senhora da Paz y se siguió hasta 2022. En ese tiempo, la dotación de personal y las pruebas variaron, lo que clasificamos en cuatro periodos: refuerzo del personal médico, de 2015 a 2017, con un médico enviado en comisión de servicios desde el Hospital Universitario Vall d'Hebron (España); personal de rutina, de 2017 a 2021, sin apoyo médico externo; tratamiento directamente observado (TDO) en la población, de 2018 a 2019, con apoyo de trabajadores comunitarios; y rastreo de contactos mejorado, de 2021 a 2022, con financiación que permitió realizar radiografías de tórax y pruebas moleculares y de aspirado gástrico gratuitas. Se evaluaron las diferencias en los contactos atendidos al mes y las pruebas y el tratamiento ofrecidos en los cuatro periodos.

**Resultados:**

En total, el programa evaluó 1978 contactos de 969 casos de referencia. La participación en el programa fue baja, aunque aumentó de manera significativa durante el periodo de TDO en la población. Solo el 16,6% (329/1978) de los contactos se sometieron a una radiografía de tórax. La confirmación microbiológica aumentó al 72,2% (26/36) tras incluir las pruebas moleculares y el 10,1% (200/1978) de los contactos recibieron tratamiento para la tuberculosis. De 457 contactos menores de 5 años, 36 (7,9%) recibieron tratamiento preventivo contra la tuberculosis. Se perdió el seguimiento de la mitad de los contactos antes de que se tomara una decisión final sobre el tratamiento.

**Conclusión:**

El rastreo de contactos aumentó el diagnóstico de la tuberculosis, aunque el compromiso con el programa fue bajo y la pérdida del seguimiento fue alta. La participación aumentó durante el TDO en la población. Se debe explorar el cribado en la población para mejorar la participación y el diagnóstico.

## ملخص

**الغرض:**

تقييم نتائج برنامج تتبع المخالطين لزيادة تشخيص مرض السل في كوبال بأنغولا، وتقديم العلاج الوقائي للفئات المعرضة للخطر.

**الطريقة:**

إن برنامج تتبع المخالطين القائم على المراكز الصحية، تم إطلاقه في مستشفى نوسا سينورا دا باز (Nossa Senhora da Paz)، في مارس/أيار عام 2015، وقمنا بمتابعة البرنامج حتى عام 2022. في ذلك الوقت، تنوع تشكيل فرق العمل، وإجراء الاختبارات، وقمنا بتصنيفها إلى أربع فترات: تعزيز فريق العمل الطبي، خلال الفترة من 2015 إلى 2017، مع طبيب منتدب من مستشفى جامعة فال ديبرون (Vall d’Hebron)، بإسبانيا؛ وفريق العمل الروتيني، خلال الفترة من 2017 إلى 2021، دون دعم طبي خارجي؛ والعلاج تحت المراقبة المباشرة من المجتمع (DOT)، خلال الفترة من 2018 إلى 2019 بدعم من العاملين بالمجتمع؛ وتتبع المخالطين المعزز، خلال الفترة من 2021 إلى 2022، بتمويل سمح بإجراء تصوير مجاني بالأشعة للصدر، واختبار جزيئي، ونضح للمعدة. قمنا بتقييم الاختلافات في المخالطين الذين تم فحصهم على مدار شهر، وتم تقديم الاختبار والعلاج خلال الفترات الأربع.

**النتائج:**

بوجه عام، قام البرنامج بتقييم 1978 مخالطًا من 969 حالة مرجعية. كانت المشاركة في البرنامج منخفضة، على الرغم من أنها زادت بشكل ملحوظ خلال فترة العلاج تحت المراقبة المباشرة من المجتمع. فقط %16.6 (329/1978) من المخالطين تم إجراء تصوير بالأشعة على الصدر لديهم. زاد التأكيد الميكروبيولوجي إلى %72.2 (26/36) بعد تضمين الاختبار الجزيئي، و%10.1 (200/1978) من المخالطين الذين تلقوا علاجًا لمرض السل. ومن بين 457 مخالطًا تقل أعمارهم عن 5 سنوات، تلقى 36 (%7.9) منهم علاجًا وقائيًا من مرض السل. وفقد نصف عدد المخالطين فرصة المتابعة قبل اتخاذ القرار النهائي بخصوص العلاج.

**الاستنتاج:**

أدى تتبع المخالطين إلى زيادة تشخيص مرض السل، على الرغم من أن المشاركة في البرنامج كانت منخفضة، وكان فقدان فرص المتابعة مرتفعًا. زادت المشاركة خلال العلاج تحت المراقبة المباشرة من المجتمع. ينبغي استكشاف فرص الفحص المجتمعي لتحسين نسب المشاركة والتشخيص.

## 摘要

**目的:**

评估接触者追踪项目的效果，以提高安哥拉库巴尔地区结核病的诊断率，并为高危人群提供预防性治疗。

**方法:**

2015 年 3 月，Nossa Senhora da Paz 医院开展了一项以卫生中心为基础的接触者追踪项目，我们一直跟踪该项目到 2022 年。我们将该项目化分为四个时期，期间配备了不同的人员和检测项目：2015-2017 年加强医务人员建设，还从西班牙瓦尔德希布伦大学附属医院借调了一名医生；2017-2021 年，只有常规工作人员，无外部医疗支持；2018-2019 年，在社区工作者的支持下，开展了社区直接观察治疗 (DOT)；在 2021-2022 年加强对接触者进行追踪，并提供资金以开展免费的胸部 X 光检查、分子检测和胃抽吸物检测。我们评估了每个月就诊的接触者的情况差异，以及在四个时期提供的检测和治疗。

**结果:**

总体而言，该项目评估了 969 例指示病例中的 1978 位接触者。该项目的参与率较低，只有在社区 DOT 期间参与率显著提高。只有 16.6% (329/1978) 的接触者进行了胸部 X 光检查。在纳入分子检测后，微生物学确诊增加到了 72.2% (26/36)，10.1% (200/1978) 的接触者接受了结核病治疗。在 457 名 5 岁以下的儿童接触者中，36 (7.9%) 名接触者接受了预防性结核病治疗。一半的接触者在做出最终治疗决定之前失访。

**结论:**

接触者追踪可提高结核病的诊断率，尽管项目的参与率较低，失访率较高。在社区 DOT 期间，参与率有所提高。因此，应探索以社区为基础的筛查工作，从而提高参与率和诊断率。

## Резюме

**Цель:**

Оценить результаты программы отслеживания контактов, направленной на повышение уровня диагностики туберкулеза в г. Кубал (Ангола), и предложить профилактическое лечение группам повышенного риска.

**Методы:**

В марте 2015 года в больнице Носса Сеньора да Паз (Nossa Senhora da Paz) была запущена программа по отслеживанию контактов в медицинских центрах. И до 2022 года велись работы по этой программе. В течение этого времени штат сотрудников и характер тестирования менялись, поэтому были выделены четыре периода: повышение квалификации медицинского персонала в 2015–2017 годах с привлечением врача из Университетской больницы Валь-д'Эброн (Испания), обычный штат сотрудников в 2017–2021 годах без внешней медицинской поддержки, лечение под непосредственным наблюдением в общине (DOT) в 2018–2019 годах с поддержкой социального работника и усиленное отслеживание контактов в 2021–2022 годах с финансированием, позволившим бесплатно проводить рентгенографию органов грудной клетки, молекулярное исследование и анализ желудочного аспирата. Проведена оценка различий в количестве контактировавших лиц, наблюдаемых в месяц, а также предлагаемых тестов и способов лечения на протяжении четырех периодов.

**Результаты:**

В целом в рамках программы было проанализировано 1978 контактов для 969 носителей заболевания. Участие в программе было незначительным, хотя его уровень существенно вырос в период проведения лечения под непосредственным наблюдением в общине. Лишь 16,6% (329/1978) контактировавших лиц была проведена рентгенография органов грудной клетки. После включения молекулярного тестирования показатель микробиологического подтверждения увеличился до 72,2% (26/36) и 10,1% (200/1978) контактировавших лиц получили лечение туберкулеза. Из 457 контактировавших лиц младше 5 лет 36 (7,9%) получили профилактическое лечение туберкулеза. Половина контактов была утеряна для проведения последующего наблюдения до принятия окончательного решения о лечении.

**Вывод:**

Отслеживание контактов способствовало увеличению количества диагностированных случаев туберкулеза, хотя вовлеченность в программу была низкой, а потери при проведении последующего наблюдения были высокими. Во время лечения под непосредственным наблюдением в общине показатели участия возросли. Следует изучить возможность проведения скринингового обследования на базе общины, чтобы повысить уровень участия и диагностики.

## Introduction

Increasing testing and diagnosis of tuberculosis (TB) is one of the main pillars of the End TB Strategy, together with rapid initiation of effective therapy and follow-up programmes.^1^ In places with a high burden of tuberculosis, recent transmission accounts for most of the tuberculosis cases. Although more and more data show that many people with tuberculosis are infected outside the house or close contact environment,^2^ evaluating close contacts of an index case increases tuberculosis diagnosis.^3^^,^^4^ In fact, a recent long-term follow-up study reported a 16-fold higher risk of developing tuberculosis in close contacts than in the general population.^5^ Although contact tracing is recommended by the World Health Organization (WHO),^6^ this strategy is not widely followed in many regions with a high burden of tuberculosis. Angola is among the 30 countries with the highest burden of tuberculosis and multidrug-resistant (MDR) tuberculosis, with an estimated incidence of 325 cases per 100 000 inhabitants and more than 100 000 incident cases a year.^7^ The national tuberculosis programme recommends screening of close contacts of a tuberculosis case, especially children and people living with human immunodeficiency virus (HIV). In reality, however, implementation of contact tracing is inadequate.^8^ As in other African countries, the main barriers to implementation are: lack of awareness of contact tracing and preventive treatment; lack and inaccessibility of diagnostic testing; socioeconomic factors; and lack of engagement of the community and public health services.^9^^,^^10^

Until 2015, no contact tracing for tuberculosis was done in the Hospital Nossa Senhora da Paz in Cubal, Angola. In March 2015, a health centre-based programme for tuberculosis contact tracing was implemented in the hospital in collaboration with the Vall d’Hebron University Hospital, Barcelona, Spain. The main objective of the programme was to increase diagnosis of tuberculosis through the systematic screening of close contacts of tuberculosis cases, and to offer preventive treatment to children younger than 5 years and contacts living with HIV. In the study, we aimed to assess the outcomes of the contact-tracing programme.

## Methods

### Setting

Cubal is a rural municipality of Benguela province of Angola with an estimated population of about 300 000 inhabitants. Hospital Nossa Senhora da Paz is a non-profit private institution which is part of the Angolan public health network. The hospital is a national reference centre for tuberculosis. The hospital has an emergency department, internal medicine department, paediatric and acute malnutrition unit, delivery room, HIV outpatient clinic, general outpatient clinic, pre- and postnatal care, and a tuberculosis sanatorium. The hospital also has radiology services (ultrasound and radiograph) and a laboratory which has recently been improved through funds from the Global Laboratory Initiative.^11^ The laboratory can perform basic blood tests, parasitological and bacterial microscopic diagnosis, molecular testing for tuberculosis, rapid diagnostic tests for HIV and CD4+ T-lymphocytes count. The tuberculosis unit of the hospital is supported by the National Plan on Tuberculosis, which provides molecular testing and antituberculosis treatment free of charge.

The Vall d’Hebron University Hospital has collaborated closely with the Hospital Nossa Senhora da Paz since 2008 through exchange of staff, training of local personnel, improvement of health facilities and research. Most of the staff working in Hospital Nossa Senhora da Paz are nurses, two of whom are in charge of the tuberculosis unit and the contact-tracing programme. Some staff had gained medical expertise working with *Médecins Sans Frontières* during the Angolan civil war (1975–2002) and through subsequent collaboration with Vall d’Hebron University Hospital personnel. For example, nursing staff have been trained on nasogastric sample collection, and laboratory staff on acid-fast bacilli smear microscopy. The hospital is funded by a sisterhood of nuns, the national general state budget and income derived from medical care. This last income is reinvested to pay some of the institution’s staff, and for medicines and other supplies for the hospital. Most people using Hospital Nossa Senhora da Paz have constrained financial resources and many live in poverty, so the costs of tests and treatments are kept to the minimum necessary to help support the institution.

### Implementation

From March 2015 until September 2022, all close contacts and household members of a sputum smear-positive tuberculosis patient were invited to attend the outpatient tuberculosis department. In January 2016, we also included contacts from sputum smear-negative tuberculosis patients. We defined contacts as per the WHO guidelines.^12^ The gender of the participants was assessed on the basis of biological attributes at the discretion of the investigators. We used the any tuberculosis symptom strategy as the initial screening, that is, asking the contacts about five symptoms suggestive of tuberculosis (cough, fever, night sweats, haemoptysis and weight loss).^12^ Hospital staff did a clinical examination of all contacts and offered them a chest radiograph and HIV testing. Staff ordered other laboratory tests such as acid-fast bacilli smear or Xpert® MTB/RIF ultra (Cepheid, Sunnyvale, United States of America) when needed. After ruling out active tuberculosis, children younger than 5 years and people living with HIV who were contacts of a patient without MDR-tuberculosis were offered preventive treatment for tuberculosis. The clinical evaluation, HIV testing and acid-fast bacilli smear testing or Xpert® MTB/RIF ultra, if considered necessary, were free of charge. Chest radiograph was free of charge only for a few months when external financial support was available. In Angola, patients frequently co-participate in their public health care, mostly for the costs of diagnostic tests and treatment. We defined a contact who was lost to follow-up as a contact who was evaluated once but who did not have any diagnostic test (acid-fast bacilli smear, Xpert® MTB/RIF ultra or radiograph) and/or never returned for a final decision on care (clinical follow-up, preventive treatment, or tuberculosis treatment). Considering costs of the chest radiograph, travel (round trip) and the materials to perform a nasogastric aspirate, we estimated that a family would face a total cost of between 5 and 10 euros per contact evaluated (about the same value in United States dollars).

**Fig. 1:**
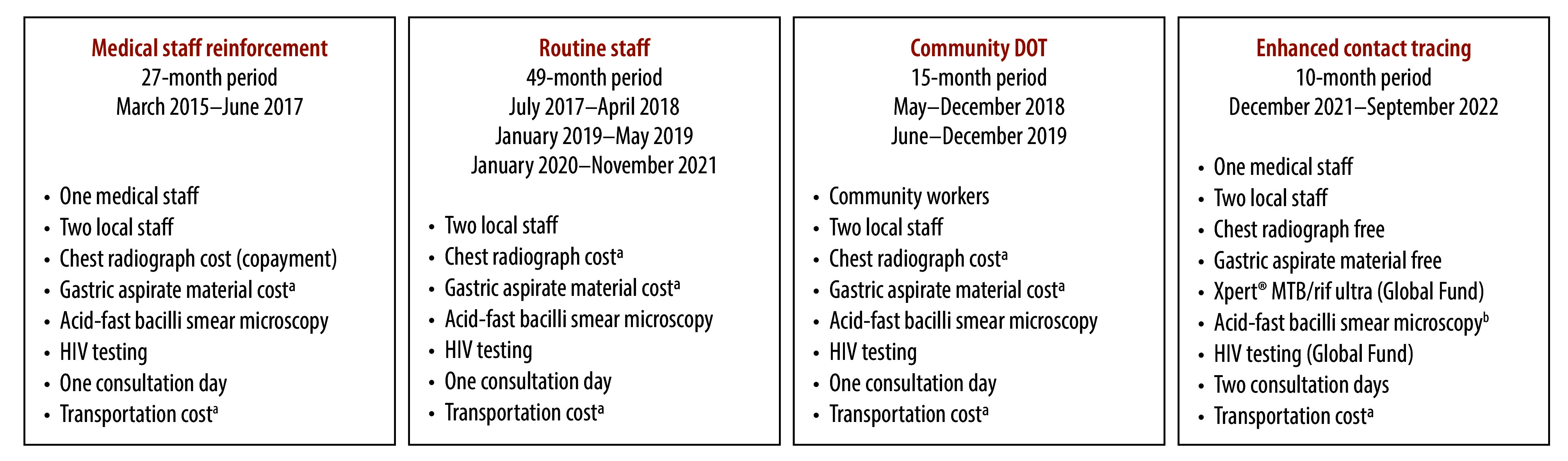
Features of each period of contact tracing, Hospital Nossa Senhora de Paz, Cubal, Angola, March 2015 to September 2022

DOT: directly observed treatment; HIV: human immunodeficiency virus.

^a^ Cost borne by the contact or families attending the contact-tracing programme.

^b^ The smear was available but not performed initially.

The contact-tracing programme was launched during the secondment of a doctor from Vall d’Hebron University Hospital’s Tropical Diseases and International Health Unit, who spent 2 years at the Hospital Nossa Senhora da Paz. We called this period the medical staff reinforcement period. After this doctor left in June 2017, local staff in charge of the tuberculosis unit took over the contact-tracing programme. We called this period the routine staff period. Because of limited staff, the contact-tracing outpatient visit was conducted once a week. In 2018 and 2019, the Hospital Nossa Senhora da Paz worked with community health workers involved in a directly observed treatment (DOT) programme (funded by the Global Fund to Fight AIDS, Tuberculosis and Malaria) to create awareness of contact tracing in the community, and refer close contacts of people with tuberculosis to the programme. We called this period the community DOT period. In 2021, Vall d’Hebron University Hospital again sent staff to Hospital Nossa Senhora da Paz to support the tuberculosis unit. In addition, in this last period Vall d’Hebron University Hospital provided funds to procure Xpert® MTB/RIF ultra and HIV tests from a Global Fund project (NFM3 project). We were therefore able to offer free chest radiographs to all contacts, implement molecular testing as the first microbiological test and test gastric aspirates free of charge. We called this period the enhanced contact-tracing period. Additionally, during this period, staff undertook contact tracing twice a week and we did not perform acid-fast bacilli smear testing initially to save laboratory personnel’s time. Fig. 1 shows the staff, tests, costs and duration of the contact-tracing periods.

### Analyses

We entered data in Excel (Microsoft, Redmond, USA) and then transferred the data to SPSS, version 19.0 (SPSS Inc., Chicago, USA) for the statistical analysis. We assessed differences in contacts seen each month, and testing and treatment offered to contacts across the four periods of the contact-tracing programme. We expressed qualitative variables as absolute numbers and percentages, and quantitative variables as means and standard deviations (SD) or medians and interquartile range (IQR) depending on the distribution. We used the χ^2^ test or Fisher exact test, when appropriate, to compare categorical variables, and the Student *t*-test for continuous variables. We considered two-tailed *P*-value of < 0.05 as statistically significant.

### Ethical considerations

We designed, implemented and reported the study in accordance with the Declaration of Helsinki, good clinical practice guidelines. The ethics committee of the Ministry of Health of Angola approved the study (nº40 C.E/MINSA.INIS/2022).

## Results

From March 2015 until September 2022, the tuberculosis unit of the Hospital Nossa Senhora da Paz diagnosed 6629 people with tuberculosis in the municipality of Cubal, of whom 695 (10.5%) had MDR-tuberculosis (Table 1). A quarter of cases were younger than 15 years. Information on the result of the acid-fast bacilli smear test was available for 686 index cases, of whom 658 (95.9%) had a positive smear.

**Table 1:** Cases of tuberculosis diagnosed at Hospital Nossa Senhora de Paz, Cubal, Angola, March 2015–September 2022

Tuberculosis cases	No. (%)								
	2015	2016	2017	2018	2019	2020	2021	2022	Total
**Total**	720	782	858	1116	952	705	820	676	6629
**MDR-tuberculosis**	91 (12.6)	56 (7.2)	60 (7.0)	85 (7.6)	117 (12.3)	92 (13.0)	99 (12.1)	85 (12.6)	695 (10.5)
**Age, in years**									
< 15	147 (20.4)	188 (24.0)	277 (32.3)	424 (38.0)	308 (32.4)	156 (22.1)	66 (8.1)	121 (17.9)	1687 (25.5)
< 5	97 (13.5)	129 (16.5)	201 (23.4)	296 (26.5)	237 (24.9)	100 (14.2)	39 (4.8)	75 (11.1)	1174 (17.7)

MDR: multidrug resistant.

During the same period, 1978 contacts from 969 (14.6%) of the 6629 index cases attended the contact-tracing clinic with a median of 1 (IQR: 1–2) contact per index case and median age of 13 (IQR: 5–29) years. Of these contacts, 1831 (92.6%) were household contacts or close relatives.

**Fig. 2:**
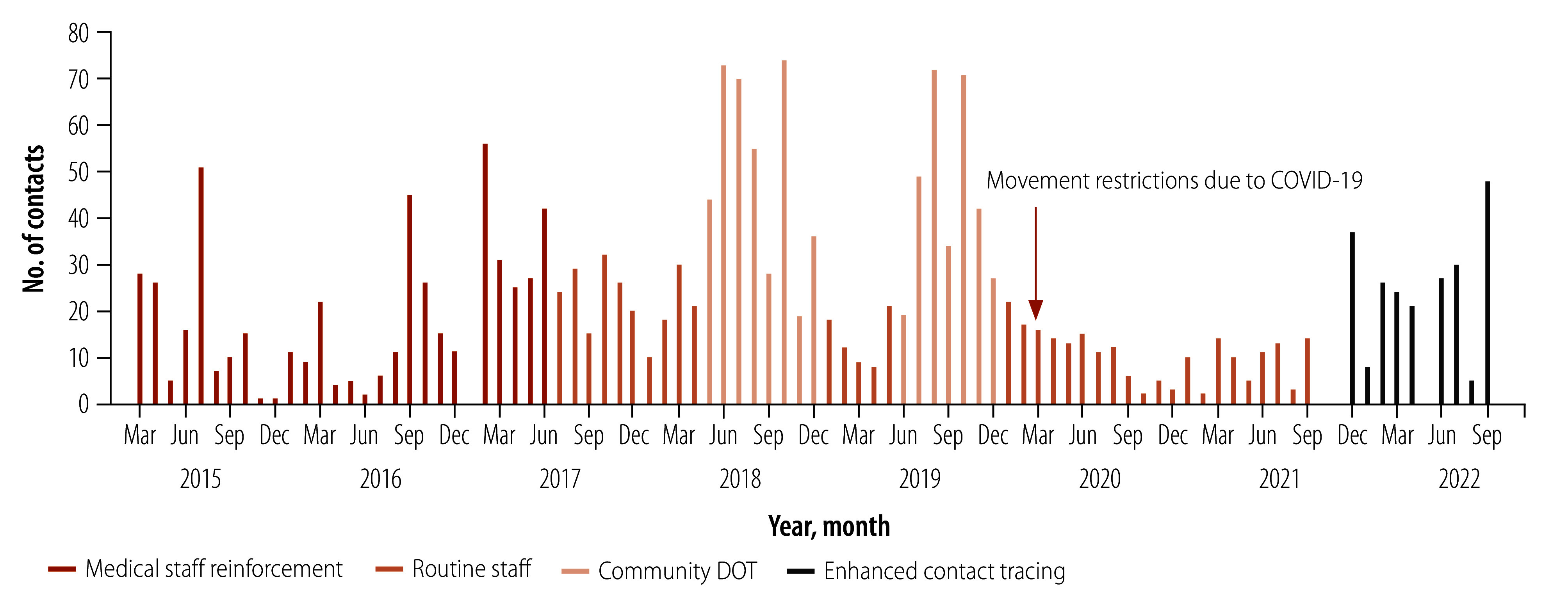
Number of contacts evaluated per month at Hospital Nossa Senhora de Paz, Cubal, Angola, March 2015 to September 2022

Overall, the tuberculosis unit evaluated an average of 22.5 (SD: 18.3) contacts per month, which more than doubled during the implementation of the community DOT period to 51.5 (SD: 20.9) contacts per month. Similarly, the number of contacts evaluated per month was significantly higher during the enhanced contact-tracing period when free tests and extra medical staff were available, and we were open for one more day of outpatient contact-tracing consultation (*P*-value: < 0.001; Table 2). Fig. 2 shows the number of contacts evaluated per month by period of the contact-tracing programme.

**Table 2:** Contact tracing results at Hospital Nossa Senhora de Paz, Cubal, Angola, by period, March 2015–September 2022

Variable	Period					*P*
	Overall *(n =* 1978)	Medical staff reinforcement (*n* = 501)	Community DOT (*n* = 688)	Routine staff (*n* = 562)	Enhanced contact tracing^a^ (*n* = 227)	
**Contacts seen/month, mean (SD)**	22.5 (18.3)	15.0 (15.6)	51.5 (20.9)	14.0 (9.2)	25.1 (13.8)	< 0.001
**Females, no. (%)**	1223 (61.8)	333 (66.5)	417 (60.6)	328 (58.4)	145 (63.9)	NA
**Children, no. (%)**						
< 5 years	564 (28.5)	194 (38.7)	158 (23.0)	139 (24.7)	73 (32.2)	< 0.001
< 15 years	1174 (59.4)	314 (62.7)	399 (58.0)	341 (60.7)	120 (52.9)	0.066
**Tuberculosis symptoms, no. (%)**	883 (44.6)	213 (42.5)	310 (45.1)	226 (40.2)	134 (59.0)	< 0.001
**Radiography, no. (%)**	329 (16.6)	155 (30.9)	17 (2.5)	29 (5.2)	128 (56.4)	< 0.001
Pathological findings	129 (39.2)	65 (41.9)	5 (29.4)	7 (24.1)	52 (40.6)	0.257
**Tested for HIV, no. (%)**	452 (22.8)	233 (46.5)	5 (0.7)	8 (1.4)	206 (90.7)	< 0.001
HIV positive	16 (3.5)	8 (3.4)	3 (60)	1 (12.5)	4 (1.9)	< 0.001
**Microbiological testing, no. (%)**						
Acid-fast bacilli smear	191 (25.5)^b^	37 (17.4)	85 (27.4)	69 (30.5)	4 (3.0)	0.004
Positive smear	85 (44.5)	7 (18.9)	25 (29.4)	19 (27.5)	2 (50.0)	0.467
Molecular testing^c^	116 (86.6)	Not done	Not done	Not done	116 (86.6)	NA
*Mycobacterium tuberculosis* detected	27 (23.3)	NA	NA	NA	27 (23.3)^d^	NA
**Started on treatment, no. (%)**	200 (10.1)	75 (15.0)	48 (7.0)	41 (7.3)	36 (15.9)	< 0.001
Microbiological confirmation	74 (37.0)	5 (6.7)	24 (50.0)	19 (46.3)	26 (72.2)	< 0.001
Children < 15 years	146 (73.0)	65 (86.7)	28 (58.3)	23 (56.1)	30 (83.3)	< 0.001
**Preventive treatment, no. (%)**						
Contacts < 5 years^e^	457 (23.1)	148 (29.5)	139 (20.2)	122 (21.7)	48 (21.1)	< 0.001
Started on preventive treatment	36 (7.9)	18 (12.2)	1 (0.7)	7 (5.7)	10 (20.8)	< 0.001
Contacts HIV-positive^f^	13 (2.9)	6 (2.6)	3 (60)	1 (12.5)	3 (1.3)	< 0.001
Started on preventive treatment	3 (23.1)	1 (16.6)	0	0	2 (66.6)	0.020
**Lost to follow-up^g^, no. (%)**	1003 (50.7)	247 (49.3)	376 (54.7)	288 (51.2)	92 (40.5)	0.003

DOT: directly observed treatment; HIV: human immunodeficiency virus; NA: not applicable; SD: standard deviation.

^a^ Medical reinforcement staff + free radiograph + free molecular testing.

^b^ Excluding 134 symptomatic contacts in the period with medical reinforcement + free of charge radiograph + molecular testing (*n* = 749).

^c^ Considering only symptomatic contacts in the period with medical reinforcement + free of charge radiograph + molecular testing (*n* = 134).

^d^ Molecular testing results for rifampicin resistance: 15 (55.6%) no resistance detected; 10 (37.0%) indeterminate; 2 (7.4%) resistance detected.

^e^ Excluding contacts treated for tuberculosis. Ninety-three (20.4%) of these 457 contacts < 5 years were contacts of an index case with multidrug-resistant tuberculosis.

^f^ Excluding contacts treated for tuberculosis. Five (38.5%) of these 13 contacts were contacts of an index case with multidrug-resistant tuberculosis.

^g^ Lost to follow-up was a contact who was visited but for whom a final decision (clinical follow-up, preventive treatment, tuberculosis treatment) could not be taken due to non-completion of diagnostic tests and/or failure to return to the outpatient clinic after the first visit.

COVID 19: coronavirus disease 2019; DOT: directly observed treatment.

Note: Included features for each period are presented in Fig. 1.

Of the 1978 contacts, 1174 (59.4%) and 564 (28.5%) were younger than 15 years and 5 years, respectively. As regards symptoms and testing, 883 (44.6%) of the 1978 contacts reported symptoms of tuberculosis and 329 (16.6%) had a chest radiograph as part of the clinical assessment. Performance of chest radiographs was highest during the enhanced contact-tracing period when 56.4% (128/227) of contacts had a radiograph taken, and was lowest during the community DOT period when only 2.5% (17/668) of contacts had a radiograph (*P*-value: < 0.001). Of the contacts with radiographs, 39.2% (129/329) had pathological findings. Of all contacts, 452 (22.8%) were tested for HIV. HIV testing was highest during the enhanced contact-tracing period when 90.7% (206/227) of contacts were tested, and lowest in the community DOT and routine staff periods when only 0.7% (5/688) and 1.4% (8/562) of contacts, respectively, were tested (*P*-value: < 0.001). Excluding the 134 symptomatic contacts from the enhanced contact-tracing period, only 191 (25.5%) of the 749 symptomatic contacts had an acid-fast bacilli smear; this test was more commonly performed in adults than children younger than 15 years (*P* -value: < 0.001). During the enhanced contact-tracing period, 86.6% (116/134) of the symptomatic contacts were tested using Xpert® MTB/RIF ultra, of whom 27 (23.3%) had tuberculosis. 

Overall, 10.1% (200/1978) of the contacts started tuberculosis treatment, 54 (27.0%) of whom were adults and 146 (73.0%) children younger than 15 years. Only 74 (37.0%) individuals overall who started tuberculosis treatment had microbiological confirmation. Microbiological confirmation of tuberculosis increased to 72.2% (26/36) of contacts who started treatment during the enhanced contact-tracing period (*P*-value: < 0.001). Similarly, 64.0% (16/25) of children younger than 5 years who started tuberculosis treatment had microbiological confirmation during the enhanced contact-tracing period. Initiation of treatment more than doubled during the two periods in which medical staff supported the contact tracing (*P*-value: < 0.001), and a significant increase in initiation of tuberculosis treatment was seen in children younger than 15 years (*P*-value: < 0.001).

Preventive treatment was started in 7.9% (36/457) of children younger than 5 years, and was significantly higher during the medical staff reinforcement period (12.2%; 18/148) and the enhanced contact-tracing period (20.8%; 10/48; *P*-value: < 0.001). Preventive treatment was started in 23.1% (3/13) of HIV-infected contacts. About half of the contacts were lost to follow-up before evaluating the complementary tests and/or taking a final decision on whether to start treatment. Significantly fewer contacts were lost to follow-up during the enhanced contact-tracing period (40.5%; 92/227), with the greatest proportion (54.7%; 376/688) lost to follow-up during the community DOT period (*P*-value: 0.003).

## Discussion

Before 2015, no tuberculosis contact tracing was performed in the municipality of Cubal. Since then, 1978 contacts have been evaluated and 200 new cases of tuberculosis have been diagnosed. Although the usefulness of contact tracing in increasing diagnosis is clear, we have struggled to fix some chronic problems. First, engagement and participation in our health centre-based contact-tracing programme has been low, with less than 15% of index cases and their contacts attending the programme. Although we promoted the contact-tracing programme both at Hospital Nossa Senhora da Paz and in the community, we did not succeed in getting patients and their close contacts to participate. Participation increased considerably thanks to the work of the community health workers, but this engagement was not maintained once these personnel had left. Overall, we believe that socioeconomic barriers contributed to the low attendance of patients and contacts. Therefore, to enhance contact tracing in a health centre-based programme, not only must community awareness be considered, but also the direct and indirect costs for close contacts of tuberculosis patients.^13^^–^^15^

Alternative strategies to overcome these socioeconomic determinants could be explored in Angola, such as contact tracing and sample collection outside health facilities (community-based contact tracing) and incentivized health care-based contact tracing. Results from a cluster randomized trial in South-Africa showed that community-based contact tracing and incentivized health care-based contact tracing are equally effective, and both prevent loss to follow-up. Furthermore, the latter strategy led to the participation of a broader group of close contacts beyond household members, which could have an added value.^16^ In addition, our results show that free testing, increasing the number of staff, and extending the number of outpatient consultation days significantly improved participation and adherence. Indeed, the lowest loss to follow-up figures were during the enhanced contact-tracing period. These later improvements resulted in better clinical assessment and counselling which probably contributed to a lower loss to follow-up.^17^ Therefore, national tuberculosis programmes should consider strengthening human resources for contact tracing.

The coronavirus disease 2019 (COVID-19) pandemic severely affected Angolan daily life. The number of tuberculosis cases diagnosed in the municipality and the contacts evaluated in the programme decreased significantly, especially during the second half of 2020 and 2021. Unfortunately, we could not do anything to avoid the impact of the pandemic. Restricted mobility, higher transportation costs (prices tripled) and the impoverishment of the population contributed to low attendance of the programme.

Lack of testing was one of the major challenges. Of particular concern was the comparatively few radiographs taken as they are especially useful for evaluating children and can help guide the decision on whether or not to start preventive treatment. During the first few years of the programme, we tried to get the patients to pay part of the costs with the rest of the cost covered through funds of Hospital Nossa Senhora da Paz. These funds have varied and have not been available since 2018. Although the number of radiographs increased significantly in the enhanced contact-tracing period, more than 40% of contacts still did not have a chest radiograph. We believe that failures in radiology services, and the distance and costs related to attending the health centre for subsequent consultations or performance of a chest radiograph could reasonably explain this figure. Given these results, portable radiograph devices and computer-aided detection could be an alternative for contact tracing in the community or in health-care centres to enhance screening and mitigate the lack of radiology specialists.^18^

Overall, HIV testing was low but a significant increase was seen during the enhanced contact-tracing period. HIV testing was performed in 22.8% (452/1978) of the contacts overall, and was significantly lower in the community DOT and routine staff periods. These low rates of testing were largely due to the frequent stock-outs of tests and shortages of tests, which were prioritized for other hospital sectors, such as HIV outpatient clinics, blood donor screening and inpatient wards.

Microbiological testing was also limited and probably related to barriers to obtaining samples from children, who were more than half of the contacts evaluated. In addition, loss to follow-up of symptomatic contacts before sample collection and stock shortages of acid-fast bacilli staining contributed to the rates of low testing. In view of this situation, from December 2021 onwards, we included molecular testing and provided materials to perform gastric aspirates free of charge. As a result of these measures, nearly nine out of 10 symptomatic contacts had microbiological tuberculosis testing; microbiological confirmation reached its maximum; the high burden of tuberculosis was confirmed among younger contacts; and first-line drug resistance could be assessed.^19^ Although the sustainability of some activities in the enhanced contact-tracing period may be compromised, the recent acceptance of stool sample molecular testing for tuberculosis diagnosis could be an easier approach to evaluate children, especially during community-based case finding.^20^ This approach would allow an initial and quicker assessment of stool and save on the costs and time of gastric or nasopharyngeal aspirates.

Our results show a higher prevalence of tuberculosis among contacts compared with data from other southern and eastern African countries.^21^^–^^23^ The high percentage of children tested in our programme, together with a possible bias due to a higher participation of symptomatic contacts, could explain these differences. In fact, the bias may be reflected in the low average number of contacts evaluated per index case, considering that the average number of household members in Angola is five. Preventive treatment for HIV-infected contacts and children younger than 5 years was low, 23.1% and 7.9%, respectively. However, such treatment was significantly higher in both periods where medical staff supported the programme, and especially during the enhanced contact-tracing period, in which radiographs and molecular testing helped rule out active disease.

The lower rates of initiation of tuberculosis treatment during the routine staff and community DOT periods could be explained by the difficulties in assessing paediatric contacts. Moreover, we believe that insufficient counselling of contacts and families, lack of isoniazid monotherapy, and lack of awareness of other drugs used for preventive treatment are limiting the start of preventive treatment for high-risk groups, especially children younger than 5 years.^24^ Therefore, continuous training and support of local staff are necessary to increase tuberculosis diagnosis and encourage the initiation of preventive treatment in high-risk groups.^25^

In conclusion, contact tracing increased tuberculosis diagnosis but many barriers to its proper implementation in Angola were encountered. Reinforcement and training of health professionals, ensuring free access to tests, reaching out to the community, considering the costs faced by families and contacts, and a commitment from the government and public health services to improve, maintain and expand these actions are important to meet the goal of ending tuberculosis in Angola.
